# Insight Into Nanoliposomes as Smart Nanocarriers for Greening the Twenty-First Century Biomedical Settings

**DOI:** 10.3389/fbioe.2020.579536

**Published:** 2020-12-15

**Authors:** K. M. Aguilar-Pérez, J. I. Avilés-Castrillo, Dora I. Medina, Roberto Parra-Saldivar, Hafiz M. N. Iqbal

**Affiliations:** Tecnologico de Monterrey, School of Engineering and Sciences, Monterrey, Mexico

**Keywords:** nanoliposomes, nanocarriers, fabrication strategies, influencing factors, drug-loaded constructs, antifungal, targeted drug delivery, biomedical applications

## Abstract

The necessity to develop more efficient, biocompatible, patient compliance, and safer treatments in biomedical settings is receiving special attention using nanotechnology as a potential platform to design new drug delivery systems (DDS). Despite the broad range of nanocarrier systems in drug delivery, lack of biocompatibility, poor penetration, low entrapment efficiency, and toxicity are significant challenges that remain to address. Such practices are even more demanding when bioactive agents are intended to be loaded on a nanocarrier system, especially for topical treatment purposes. For the aforesaid reasons, the search for more efficient nano-vesicular systems, such as nanoliposomes, with a high biocompatibility index and controlled releases has increased considerably in the past few decades. Owing to the stratum corneum layer barrier of the skin, the in-practice conventional/conformist drug delivery methods are inefficient, and the effect of the administered therapeutic cues is limited. The current advancement at the nanoscale has transformed the drug delivery sector. Nanoliposomes, as robust nanocarriers, are becoming popular for biomedical applications because of safety, patient compliance, and quick action. Herein, we reviewed state-of-the-art nanoliposomes as a smart and sophisticated drug delivery approach. Following a brief introduction, the drug delivery mechanism of nanoliposomes is discussed with suitable examples for the treatment of numerous diseases with a brief emphasis on fungal infections. The latter half of the work is focused on the applied perspective and clinical translation of nanoliposomes. Furthermore, a detailed overview of clinical applications and future perspectives has been included in this review.

## Introduction—Problem Statement and Opportunities

Conventional drug delivery systems (DDS) are used to deliver therapeutic molecules into the human body either by oral consumption, injection, or topical administration. These systems were extensively in practice and accepted as convenient in terms of ease in administration. However, disadvantages are governed mainly because of the lack of compatibility at requisite level, poor biodistribution, burst or disrupted release, and low accuracy to reach the target sites in a sustainable and sophisticated manner (Dang and Guan, 2020). There is a dire need for highly effective and less/non-toxic alternatives to treat existing and emerging diseases. Besides, this has also provoked the medical sector authorities to search for robust therapeutic agents and new ways to increase the efficacy of traditional drug delivery agents (Taboada and Grooters, 2008). Scientists have engineered several types of nanocarrier mechanisms, such as solid lipid nanoparticles (SLN), liposomes, polymeric micelles, metallic nanoparticles (MNPs), spanlastics, nanoemulsions, nanoliposomes, among others (Taboada and Grooters, 2008; Elsherif et al., 2017; Huang et al., 2017; Zamani et al., 2018; Permana et al., 2019; Yang et al., 2020), either to develop new drug formulations or improve the existing ones. Many of these nano-systems are capable of inducing/imparting pharmacological activities, enhance drug dynamism, and improve physical stability to attain controlled release characteristics (Haury et al., 2017). Furthermore, the newer nanomedicines with a topical approach can counteract the issues associated with conventional and systemic therapy for the treatment of infections and, at the same time, reducing the high-cost impact and minimizing long-term side effects (Gupta et al., 2017).

Nanoliposomes have been referred to as nanoscale bilayer lipid vesicles since the term liposome is a broad definition, including various types of vesicles with average size up to several micrometers (Mozafari and Mortazavi, 2005; Patil and Jadhav, 2014). Nanoliposomes present a greater surface area and have acceptable stability profile to preserve their size within nanometric scales, e.g., as small as 20–100 nm (small liposomes) and >100 nm (large liposomes) (Khorasani et al., 2018). These carriers are mainly composed of lipids and phospholipids. However, some contain other molecules, such as carbohydrates, antioxidants, proteins, or sterols in their structure (Mozafari and Khosravi-Darani, 2007). Due to their amphiphilic nature, they have the potential to entrap and release a massive range of hydrophilic and hydrophobic compounds simultaneously providing a combined benefit. Additionally, their characteristic bilayer structure is highly compatible with the skin surface, allowing them to act as penetration enhancers of bioactive compounds toward targeted sites (Farghaly et al., 2017). Compared with other nano DDS, nanoliposomes have the advantage of being produced using natural and inexpensive ingredients on an industrial scale (Demirci et al., 2017). This advantage, together with biocompatibility and biodegradability, make nanoliposomes very fascinating as “smart” drug delivery vehicles. Comparative overview of advantages and disadvantages of liposomes and nanoliposomes are summarized in Table 1. Keeping in mind the given attributes of liposome and nanoliposomes in Table 1, continuous research to enhance the already known properties of nanoliposomes keeps constant among research groups by conferring new structural characteristics throughout possible mechanisms of synthesis and surface modification to improve their potentialities, stability, and shelf-life (Jin et al., 2018). The present review focuses on the recent development in nanoliposome-based DDS with a brief emphasis on fungal infections. We also summarized fabrication techniques and several influencing factors that can significantly affect the overall fabrication and performance of nanoliposome-based DDS. Given, the current state of the art, including advantages and limitations, and a general overview of other novel nanostructured carriers that also exhibit important features for biomedical applications are discussed with suitable examples.

**Table 1:** Comparative overview of advantages and disadvantages of liposomes and nanoliposomes.

**Advantages**	**Disadvantages**
**LIPOSOMES**	
✓ Entrapment of hydrophilic and hydrophobic compounds separated or simultaneously.	✗ Reduction in encapsulation efficiency due to size enlargement
✓ The increase in number of layers (e.g., kinetic constraints) may be beneficial to prevents or delays the release of active molecules.	✗ Higher physical instability during storage.
✓ Made of natural ingredients	✗ Susceptibility to fast clearance from the bloodstream
✓ Simple fabrication process	✗ Drug leakage
✓ Possibility of surface functionalization	✗ Higher susceptibility to be capture by RES
✓ Cost-effectiveness	✗ Reduced bioavailability compared to nanoliposomes
**NANOLIPOSOMES**	
✓ Entrapment of hydrophilic and hydrophobic compounds separated or simultaneously.	✗ Manufacturing process usually involves mechanical energy (e.g., sonication, homogenization, microfluidization, etc.) that may degrade the lipid structure.
✓ Reduced toxicity and side-effects	✗ Aggregation and coalescence can occur due to stronger electrostatic interactions.
✓ Greater stability when incorporated into real products	✗ More clinical trials are still necessary
✓ Higher surface area-to-volume ratio	✗ In some cases, the use of surfactants as stabilizers is needed.
✓ Better solubility and accurate targeting	✗ Reduced drug storage capacities
✓ Delayed body clearance and better suitability for chemotherapeutics delivery	✗ The *in vivo* fate is still not fully understood

## Nanostructured Systems—A Drive Toward Optimum Performance

Nanostructured DDS can upgrade the features of traditional drug administration within the biomedical field. The use of nano lipid carriers is considered a safe route of drug administration (Haury et al., 2017; de Matos et al., 2019). Notwithstanding the considerable variety of nanostructured systems that have been used for biomedical purposes, there are still several challenges to overcome. For instance, some studies have reported the toxicity behavior of MNPs in the central nervous system (Sawicki et al., 2019). In contrast, bio-ceramic nanoparticles have been successfully applied for prosthesis, implants, and tissue regeneration (Thian et al., 2017). Nevertheless, their rate of clearance from the body by bloodstream varies from material to material and leads to their accumulation in body organs or mononuclear phagocytic system (Singh et al., 2017). Couple with this, the side effects and data related to toxicity effects may vary when the administration route, fabrication process, and functionalization agents are considered. Nanoliposomes have been investigated and incorporated into medicines for different purposes. The Food and Drug Administration (FDA) had approved their use in cancer therapy, vaccine delivery, fungal and microbial infections, analgesics, among others resulting in their high biocompatibility with the human body and potential pharmacokinetic profile (Inglut et al., 2020). Therefore, they can enhance the pharmacokinetic and pharmacodynamic profiles of the therapeutic payload, facilitate controlled and sustained release of the loaded drugs (Mohammadabadi and Mozafari, 2018).

### Nanoliposomes

**Figure 1:**
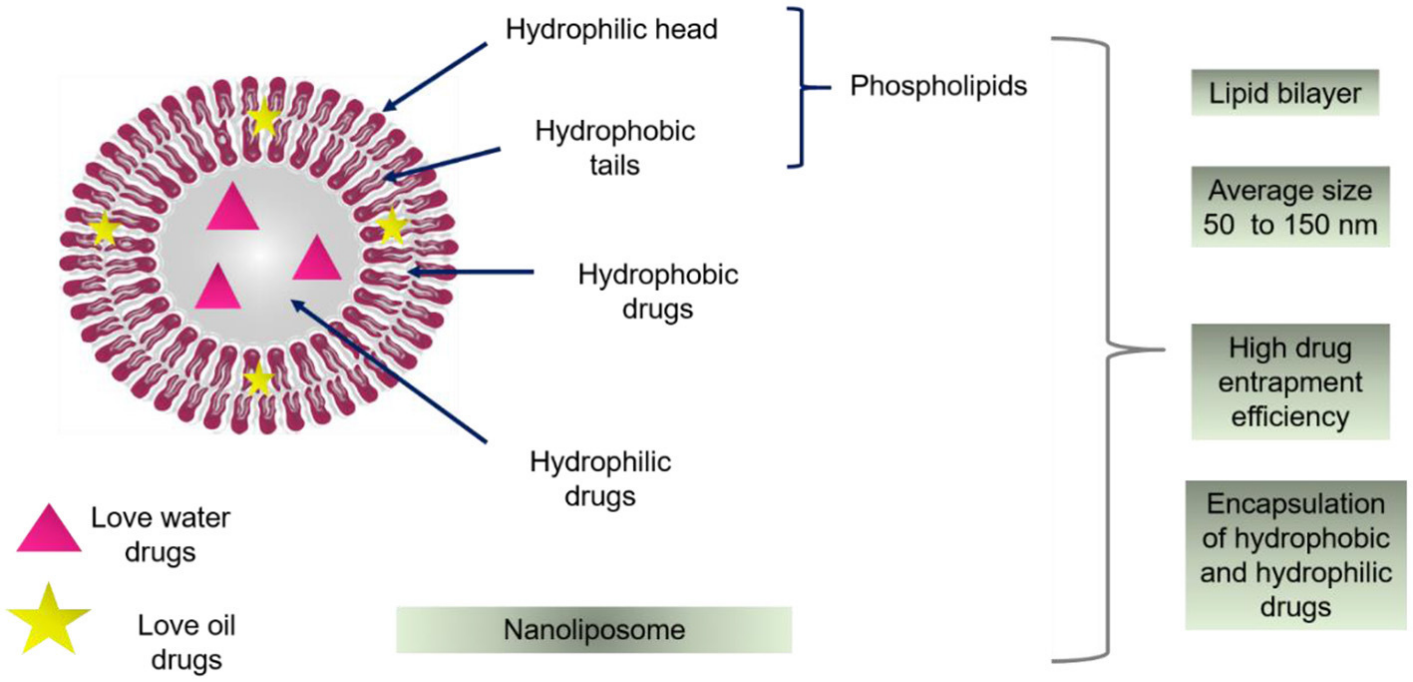
Schematic diagram of the bilayer structure of nanoliposomes with the representation of their amphiphilic structure for the entrapment of hydrophobic and hydrophilic drugs. On the right, some of the remarkable characteristics of these systems are listed. The liposomal structure is mainly composed of phospholipids such as phosphatidylcholine, phosphatidylserine, or phosphatidylethanolamine. Nonetheless, the addition of cholesterol in the liposomal formulation is very common with the purpose of providing stability and rigidity in the lipid membrane.

**Figure 2:**
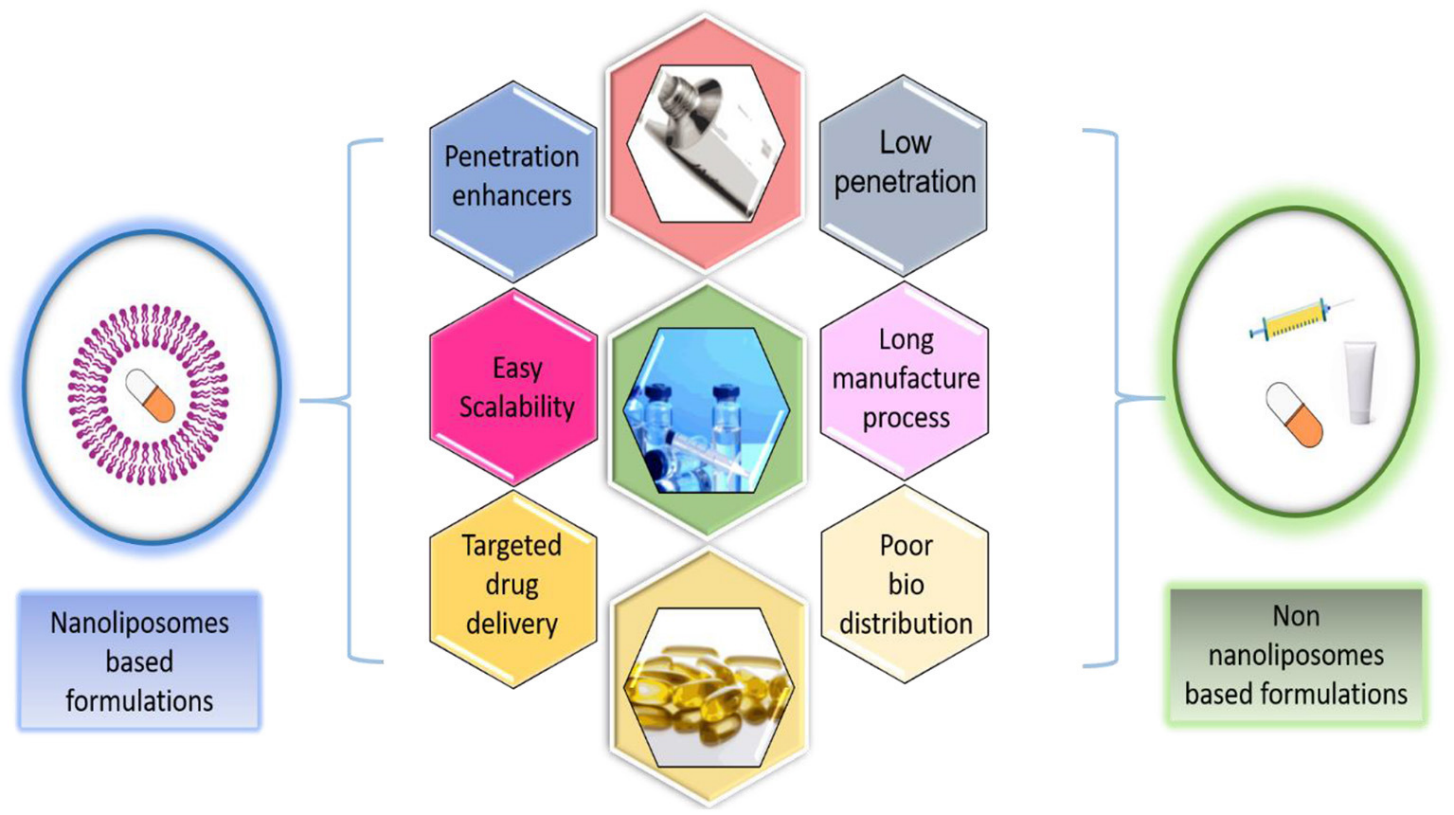
Advantages of nanoliposomes based formulations respect to non-nanoliposomes based formulations for oral, topical, and intramuscular drug administration.

Broadly speaking, the nanoliposomes are defined as bilayer lipid vesicles, as shown in Figure 1, which possess and maintain nanometric size ranges during storage and applications (Khorasani et al., 2018). Due to their bilayer structure, composed of lipidic and aqueous sections, these nano-systems can encapsulate hydrophilic and hydrophobic compounds individually or at the same time. Notwithstanding the potentialities of these nano-systems as drug delivery carriers, low physical stability, high sensitivity to temperature, and pH variations are significant challenges to overcome when commercial use is intended. However, numerous investigations have been reported the surface modification to improve stability and storage (Milani et al., 2019). In consequence of enhanced stability and targeting, the amount of entrapped material is less than the amount required without encapsulation. This may be helpful when working with high-cost bioactive compounds. Additionally, the use of natural and inexpensive ingredients (e.g., soy, egg yolk, sunflower, milk) for nanoliposome preparation is possible, thus, optimizing the cost-effectiveness of the final product (Khosravi-Darani and Mozafari, 2010). Because of these unique properties, numerous clinical trials have revealed that nanoliposomes are great candidates for varied delivery systems, such as anti-cancer, anti-fungal, and anti-biotic drugs, the delivery of gene medicines, and the delivery of anesthetics and anti-inflammatory drugs (Allen and Cullis, 2013). Advantages of nanoliposomes based formulations with respect to non-nanoliposomes based formulations for oral, topical, and intramuscular drug administration are presented in Figure 2.

**Figure 3:**
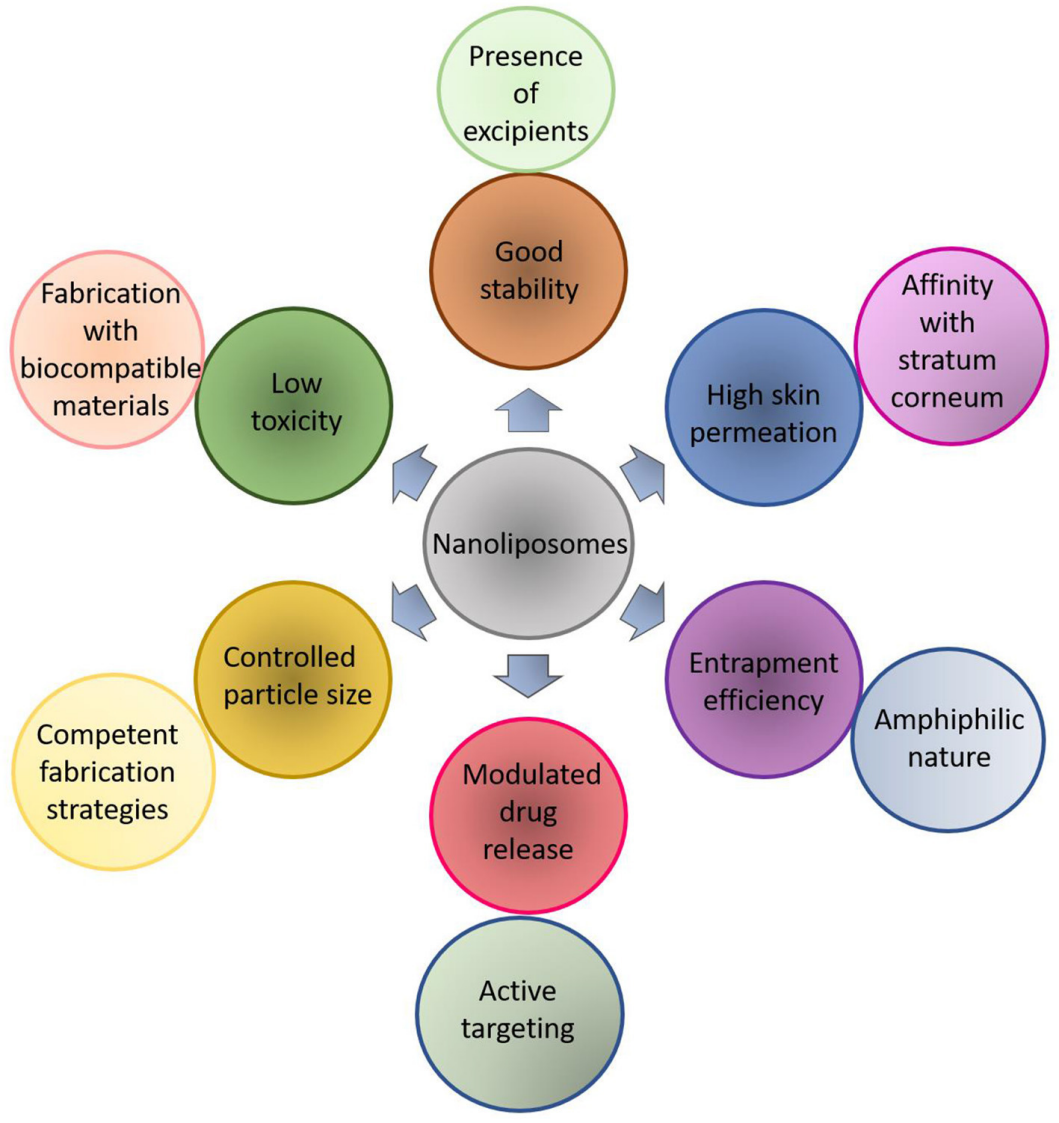
Schematic representation of multi-functional characteristics of drug loading into nanoliposomes as a competent model for biomedical applications.

Nanoliposomes have also been combined with other clinical techniques to improve their mechanism of action. Gelfuso et al. (2020) tested the effectiveness of voriconazole based nanoliposomes along with iontophoresis for the treatment of fungal keratitis. The system was evaluated against *Candida glabrata* culture, the minimal inhibitory concentration (MIC) for voriconazole in the presence/absence of iontophoresis on *C. glabrata* was 0.14 ± 0 and 0.28 ± 0 μg/ml. The liposomal formulations did not present an excellent advantage for iontophoretic delivery at a current density of 2 mA/cm^2^. Besides, the morphological analyses performed by Transmission Electronic Microscopy (TEM) displayed an oval shape close to 100 nm. These results confirmed the excellent stability and the strong capability of nanoliposomes for voriconazole passive delivery over commercial voriconazole medicine. Both carriers have been successfully applied for biomedical proposes in view of their drug delivery mechanism and release behavior (Khorasani et al., 2018; Subramani and Ganapathyswamy, 2020). Figure 3 represents the multi-functional characteristics of drug loading into nanoliposomes as a competent model for biomedical applications.

## Fabrication Strategies—Processing and Workflow

### Thin-Film Hydratio—Sonication Method

This methodology, also known as Bangham method (Bangham et al., 1965), is the most implemented to synthesize conventional nanoliposomes. A mixture of phospholipids is dissolved in a polar solvent (e.g., ethanol) with the hydrophobic drugs. Afterward, the solvent is evaporated (either via rotary evaporator or sample concentrator) above the transition temperature of phospholipid. Then, a film is formed at the bottom of the flask and kept drying under a vacuum desiccator for 24 h or more to remove traces of organic solvents before hydration. The hydration is carried out under stirring in the presence of distilled water or buffer solution such as phosphate buffer saline (PBS) or 4-(2-hydroxyethyl)-1-piperazineethanesulfonic acid (HEPES) (Gallez et al., 2020). Subsequently, the mixture is sonicated either through a bath or probe sonicator to reduce the vesicle size and to homogenize the sample.

### Ethanol Injection Technique

This technique was described by Batzri and Korn (1973). An ethanol solution of phospholipids is injected under controlled conditions considering pump flow rate, stirring intensity, and injection temperature (above lipid transition temperature) into an aqueous phase. Subsequently, the solution remains under mechanical stirring on a magnetic stirrer or by rotary evaporation at room temperature under reduced pressure to remove the traces of solvent (Toniazzo et al., 2017; Hammoud et al., 2020).

### Reverse Phase Evaporation Method

In this method, the lipid mixtures are dissolved in an organic solvent and solubilized with the aid of an ultrasonic bath. Then, a liquid solution, either water or buffer with stabilizers, is added to the mixture. Following that, the solvent is evaporated under reduced pressure by a rotary evaporator to promote a dense gel formation. An excess of the liquid solution is added to evaporate remains of organic solvent. The final formulation is submitted to dialysis, sonication, or centrifugation to homogenize the particle size. Nitrogen atmospheres can be implemented to purge the system to protect the lipid mixtures from degradation (Shi and Qi, 2018; da Rosa et al., 2019).

### Supercritical Fluid Technology

A supercritical fluid is defined as a compound at temperature and pressure above their critical point exhibits properties of liquids such as density and gases such as compressibility. CO_2_ is the most regularly used supercritical fluid mainly due to its low price and other characteristics, including low critical temperature and pressure (31.1°C and 73.6 bar) and recyclability (Moreno et al., 2019). Supercritical fluid technology has been developed to reduce the use of organic solvents such as chloroform, ether, or methanol during the preparation of nanoliposomes due to their harmful risk to the environment and human health. Moreover, these substances result in challenging to separate by using conventional synthesis (Zhang et al., 2012). In Supercritical fluid technologies, the use of organic solvents is not always eluded. Still, whenever their use becomes necessary, they usually have a lower toxicity index than the previously mentioned solvents (Lesoin et al., 2011).

The most common supercritical fluid technologies that involve the fabrication of nanoliposomes are supercritical antisolvent (SAS) and rapid expansion of supercritical solutions (RESS). SAS implies the use of an organic liquid co-solvent which already contains the phospholipid mixture. It must be miscible in the presence of the supercritical fluid, which proceeds as an anti-solvent to precipitate the lipid material, promoting nanoparticles' formation (Gupta and Xie, 2018; Schwartz et al., 2018). In the RESS procedure, solutes are dissolved at high pressure in the supercritical fluid, posteriorly the solution is decompressed with the aid of a nozzle and then precipitated by rapid expansion with the purpose to enable rapid nucleation. Subsequently, adequate particle formation, in this case, supercritical CO_2_ acts as a solvent (Debenedetti et al., 1993; Gomes et al., 2018).

### Supercritical Assisted Liposome Formation (SuperLip)

This synthesis methodology belongs to the dense gas technologies. It consists in the use of a dense gas such as carbon dioxide (CO_2_) to enhance the mixing between the organic phase (phospholipids and ethanol) and water and to remove the traces of ethanol from liposomes suspension. The organic mixture is pumped in a static mixer with CO_2_ under controlled pressure and temperature, usually 100 bar and 40°C to obtain a gas-expanded solution. The resulting ethanol expanded solution is pumped with a water phase into a high-pressure chamber. Simultaneously, the water is sprayed throughout a nozzle. Finally, ethanol is separated from vesicles and water suspension and recovered in a separator by CO_2_ flushing out from the chamber under pressure at room temperature (Ciaglia et al., 2019; Trucillo et al., 2019). This technique's feasibility for the synthesis of nanoliposomes allows getting an adequate control of particle size and distribution and high entrapment efficiency (up to 84%) (Trucillo et al., 2020).

### Depressurization of an Expanded Liquid Organic Solution (DELOS-SUSP)

This technique is performed by adding a sample containing lipids and organic solvent into a vessel at working temperature (T_w_) and atmospheric pressure (P_atm_). The expansion of the lipid is carried out by adding a large amount of CO_2_ to obtain an expanded solution, considering that the lipids must be soluble in the CO_2_-expanded solvent to guarantee the formation of a single-phase inside the high-pressure chamber until reach the working pressure (P_w_). Finally, depressurization of CO_2_- the expanded solution is done over a flow of aqueous phase from (P_w_) to (P_atm_) containing a surfactant whenever it is necessary to provide better uniformity to the vesicles (Elizondo et al., 2011). In this final step, a flow of N_2_ at P_w_ is used to push down the CO_2_-expanded solution and to keep the pressure inside the vessel constant (Grimaldi et al., 2016).

### Particles From Gas Saturated Solution (PGSS)

This fabrication technique consists of two steps. The first step involves the saturation of a solute with CO_2_ in a mixing container at high pressures. The second step refers to the expansion of the gas saturated solution with the aid of a nozzle at (P_atm_). The formation of the material occurs during the development due to the fast reduction in temperature (Joule-Thompson effect), producing particle formation by solidifying the material. This technique has been used for the encapsulation of bioactive compounds in liposomes. However, entrapment efficiency reported is low compared to other techniques, such as the thin-film hydration method (Varona et al., 2011). Moreover, another study reported the development of high-quality vesicles, enough dispersion, and storage stability for up to 4 weeks (Zhao and Temelli, 2015).

### Depressurization of an Expanded Solution Into Aqueous Media (DESAM)

In this technique, the hydration process is performed by depressurizing an expanded solution into an aqueous media via a nozzle. A mixture of lipids in an organic solvent is injected into an expansion chamber. The operating conditions are carried out at moderate temperatures and pressures below 60 bar. The expanded lipid solution is reached by pressurization through the addition of dense gas, and it is further heated into an aqueous media. The controlled release of the developed lipid solution is performed when the pressure is maintained by adding dense gas. The organic solvent is washed from the system. This ensures minimal residual solvent and can be separated and recycle with the gas leaving the system (Meure et al., 2009; Campardelli et al., 2015).

### Heating Method

A new technique for the fast fabrication of nanoliposomes avoids using hazardous solvents developed by Mozafari et al. (2002) implies the use of a single vessel in the absence of solvents or detergents. The phospholipids and excipients are hydrated under an inert atmosphere for 1–2 h in an aqueous medium. Therefore, the ingredients are put through mechanical stirring after the addition of a polyol, such as glycerol, which acts as a cosolvent or dispersant at a temperature up to 120°C for 30 min to ensure a proper ingredients distribution in the aqueous medium. Once the ingredients are uniformly dispersed, drug compounds can be added either at a high or lower temperature, depending on their heat sensitivity (Danaei et al., 2018b).

### Mozafari Method

This method belongs to one of the modern techniques for the synthesis of nanoliposomes developed. Given most of the current processes for the fabrication of nanoliposomes that require either solvents, high shear mixers, or pressurization. To overcome these drawbacks, Colas et al. (2007) introduced an improved version of the heating method for the encapsulation of nisin called Mozafari method. The authors proposed that nanoliposomes' synthesis can be carried out in a home-made glass vessel designed by Mozafari. This type of glass-bottle was developed to enhance the methodology's efficiency since the multiple turbulences contained in a single vessel enable to function as seven vessels simultaneously. Thus, having seven as the total number of turbulences. This method allows the manufacturing of nanoliposomes in a single step without employing solvents, detergents, and the need for pre-hydration. The liposomal ingredients are added in a preheated mixture that contains the active compound that is pretended to encapsulate and a polyol. Then the mixture is heated while stirring under a nitrogen atmosphere. If it is intended to incorporate cholesterol into the formulation, this must be added in the aqueous phase while stirring at elevated temperatures under a nitrogen atmosphere before mixing the other phospholipid components. Finally, the nanoliposomes suspension is subjected under an inert atmosphere above the lipid transition temperature to allow sample annealing and stabilization, as mentioned in previous work (Mozafari, 2010). Table 2 summarizes the advantages and disadvantages of each nanoliposome fabrication technique. Additionally, recent reports that discuss the fabrication strategies to engineer nanoliposomes for drug delivery purposes are outlined in Table 3.

**Table 2:** Advantages and disadvantages of various fabrication techniques used for the development of nanoliposomes.

**Fabrication technique**	**Advantages**	**Disadvantages**
Thin film hydration—sonication method	Economic Easy to perform	Use of organic solvents Exposure to mechanical stress
Ethanol injection	Simple procedure Good stability profile	Low encapsulation efficiency Time consuming
Reverse phase evaporation	Simple design Decent percentage of encapsulation efficiency	Large amount of organic solvent
SAS	Low organic solvent consumption	Use of sophisticate machinery, expensive
RESS	Absence of liquid organic solvents Mild processing temperatures	Implementation of complex apparatus Requirement of high pressures
SuperLip	Control of particle size High encapsulation efficiency	Use of high pressures Use of CO_2_
DELOS-SUSP	Easy scale up production Uniform particle size	Low entrapment efficiency Use of solvent and necessity to produce an expanded solution
PGSS	High encapsulation efficiency Larger particle sizes	Use of expensive instrumentation Low stability
DESAM	Fast and simple for bulk nanoliposome formation Alternative to current gas dense technologies	Use of organic solvent Multi step procedure
Heating method	Avoid the use of toxic solvents and detergents	Use of inert atmospheres (Ar or N_2_)
Mozafari method	Easy to perform Brief protocol for industrial scalability	Use of inert atmospheres and polyols

**Table 3:** Recent reports about fabrication strategies of nanoliposomes applied to substances of interest in drug delivery.

**Synthesis technique**	**Encapsulated agent**	**Objective**	**Results**	**References**
Thin-film hydration—sonication	calothrixin B	To test anticancer activity against lung and breast cell lines A549 and MCF-7	High entrapment efficiency, Control size distribution, increased stability	Yingyuad et al., 2018
Ethanol injection	Black seed oil (Nigella sativa)	To enhance oral bioavailability and improve therapeutic activity in small animal studies of analgesia	Improvement of analgesic activity and oral bioavailability. Sucrose and cholesterol exhibited to improve the encapsulation efficiency of black seed oil.	Rushmi et al., 2017
Reverse phase evaporation	Pomegranate extract	To carry an efficient amount of pomegranate extract to sperm via lecithin nanoliposome to protect sperm against lipid peroxidation	Protection of ram sperm during cryopreservation without adverse effects. Pomegranate formulation improved the quality of ram semen after thawing	Mehdipour et al., 2017
Supercritical fluid technology	Melatonin	To load melatonin in nanoliposomes as a delivery system in order to increase its oral bioavailability	Uniform size distribution. Slow release feature in early digestive stages and more thorough characteristics in later stages of simulated digestion	Zhang et al., 2017
Supercritical assisted Liposome formation (SuperLip)	Amoxicillin	Encapsulation of an antimicrobial agent for intravenous application	Inhibition growth of *E. coli* bacteria Encapsulation efficiency up to 84%	Trucillo et al., 2020
Depressurization of an Expanded Liquid Organic Solution (DELOS)	α-Galactosidase-A	To produce protein-nanoliposome for the treatment of Lysosomal storage disorders (LSD)	Enhanced enzymatic activity and intracellular penetration. Entrapment efficiency of 40%	Cabrera et al., 2016
Particles from gas saturated solution PGSS	–	To investigate the effect of pressure, depressurization rate and temperature on the characteristics of the final formulation	Quality of the vesicles depends on the dispersion of the phospholipid molecules prior to their reorganization during the processing	Zhao and Temelli, 2015
Depressurization of an Expanded Solution into Aqueous Media (DESAM)	–	To design and validate a new process for bulk liposome formation	Range size from 50 to 200 nm Polydispersity index below 0.29	Meure et al., 2009
Heating method	Plasmid DNA	To prepare anionic nanoliposomes without using any volatile organic solvent or detergent in order to test their morphology, stability and DNA incorporation efficiency	Good reproducibility, long-term stability and potential nano liposome production in large quantities.	Mozafari et al., 2002
Mozafari method	Polyunsaturated fatty acids (PUFAs): docosahexaenoic acid (DHA) and eicosapentaenoic acid (EPA)	To investigate the oxidation of bulk DHA and EPA incorporated into liposomes during cold (4 °C) storage	Enhancement of the oxidative stability of DHA and EPA in aqueous media when compared with bulk systems	Rasti et al., 2012

## Influencing Factors that Affect the Nanoliposomes Performance

### Permeability/Penetration Capacity

Nanoliposomes raise targeting of drug penetration of active ingredients through vesicle adsorption onto the skin surface by the interaction of lipids part of nanoliposomes with the stratum corneum. The lipid bilayer of nanoliposomes can fuse with other bilayers due to its resemblance to the biological membrane, which simplifies the penetration into the epidermal barrier and helps in the transport of the core therapeutic material compared to other nano DDS (Siepmann et al., 2012; Arshad et al., 2020). The passage of nanoliposomes through the horny layer is enhanced by the occlusive effect that increases their permeability (Hofland et al., 1995; Touti et al., 2020). The occlusive effect refers to an increase of hydration in the stratum corneum in the presence of water, affecting percutaneous adsorption by amending segregation between the surface chemical and the skin (Foldvari et al., 1990). This passage can be favored by the active principle's affinity for the horny layer and promotes the increased penetration of lipid-soluble. The topical-based drug formulation that contains fats and/or polymers oils may also generate occlusive effects, becoming suitable for pharmaceutical and cosmetic applications (Zhai and Maibach, 2002; Van Tran et al., 2019).

Nanoliposomes systems have received particular attention for drug delivery applications due to their bilayer structure that affords the substantial capability to entrap hydrophobic and hydrophilic molecules acting as penetration enhancers. Size is an essential factor to bear in mind since smaller sizes lead to larger surface areas and subsequently to greater reactivity and control the drug's release kinetics (Samadi et al., 2020). According to Sakdiset et al. (2018), composition and design are essential factors to consider for developing efficient nanoliposome with high skin permeation and improved performance. The research group found that 1,2-di- palmitoyl-sn-glycero-3-phosphoglycerol, sodium salt (DPPG) can be a promising phospholipid candidate for nanoliposome formulations with high skin penetration-enhancing effects. They tested the mechanism of interaction of empty nanoliposomes and entrapped caffeine where DPPG phospholipid and nanoliposome vesicles had a combined effect of disrupting the stratum corneum lipid barrier to carry both in the formulation through the skin. Pseudo ceramide loaded nanoliposomes were synthesized, and their role in skin barrier functions was investigated by Kim et al. (2019). The nanoliposomes functionalized like skin constituents were prepared using pseudo ceramides, PO3C, PO6C, PO9C, and loaded with baicalein. The *in vivo* skin permeation results showed that the nanoliposome formulation carried baicalein well and effectively penetrated the skin. The use of pseudo ceramides not only passed the skin barrier but also effectively transmitted the weakly soluble drug, baicalein, which demonstrated the use of nanoliposomes, as functional carriers, that effectively transmit the poorly soluble drug baicalein to the skin.

### Drug Loading Capacity

The entrapment efficiency (EE) and loading capacity (LC) are crucial parameters for promising applications of nanoliposomes due to the necessity of produce formulations with the desired payload with minimal drug loss (Drummond et al., 2010). Among the reasons for medical applications of nanoliposomes is the effectiveness to load acceptable quantities of drugs needed to achieve therapeutic efficacy. However, it must consider several factors that may affect the performance of nanoliposomes as drug-loaded carriers (Zucker et al., 2009), developed a model based on loaded conditions of liposomes and nanoliposomes drugs'. They found that the most critical condition that affects loading capacity is the initial drug/lipid mole ratio. Precisely, when it is too high with values above 0.95, low loading capacity is displayed due to excess drug that exceeds the liposomal loading capacity, which entails overloading damages in the lipid membrane leading to a lower final drug/lipid mole ratio. Moreover, some other factors, such as solubility, pH, drug properties, temperature, and loading conditions, must be reviewed for suitable drug-loaded liposome formulations and to improve their development for clinical applications.

### Surface Modification

A drug molecule's therapeutic potential depends on its availability at the target site at the requisite amount and for the required duration. Besides, it is essential to minimize drug exposure to non-target tissues to avoid potential side effects. The use of nano DDS, such as nanoliposomes, has helped in improving drug efficacy and safety by modifying the pharmacokinetic properties, for instance, distribution, absorption, and elimination of the drug (Mozetič, 2019). Their small particle size range enables systemic administration because the smallest blood capillaries are 10–20 μm in diameter (Zamani et al., 2018). Further, carriers in this size range could be used for targeted delivery of different types of therapeutic payloads to specific organs and tissues (Moku et al., 2019). In recent years the problem of phagocytic removal of nanoparticles has been solved by surface modification of nanoparticles. The surface modification protected nanoparticles from being phagocytosed and removed from the blood vascular system after intravenous injections (Mahapatro and Singh, 2011). However, nanoliposomes are like biological membranes and are more suitable for cellular absorption. It has been reported that phospholipid bilayer maybe suffers oxidation damage during storage conditions (Islam Shishir et al., 2019). The surface modification of conventional nanoliposomes can enhance stability under storage conditions, improve phospholipid bilayer permeation, and protect the loaded drug (Sperling and Parak, 2010). Karim et al. (2020) reported that surface decoration of neohesperidin-loaded nanoliposome using chitosan and pectin could improve stability and controlled release. The results confirmed good encapsulation efficiency (>90%), the particle size of 79.50 ± 0.72 with zeta potential values of −29.63 ± 0.81. The modified nanoliposomes coated with chitosan (CH-NH-NL) and pectin (P-CH-NH-NL) were compared to conventional nanoliposomes loaded neohesperidin (NH-NL). Even though all nanoliposomal formulations exhibited mucoadhesion ability, the modified samples showed the highest mucin adsorption percentage and were more effective in preserving neohesperidin. Storage results unveiled that nanoliposomal systems can be stable for 30 days at 4°C in the dark condition. However, throughout the storage study, the particle size of NH-NL was higher than that of CH-NH-NL and P-CH-NH-NL. As a result, the decoration of nanoliposomes can be a promising way to improve the physicochemical stability, controlled release behavior, and mucoadhesion ability.

Similarly, modified nanoliposomes have presented potentialities as an ocular delivery system to treat glaucoma. Jin et al. (2018) investigated D-alpha-tocopheryl poly (ethylene glycol 1000) succinate (TPGS) modified nanoliposomes for brinzolamide (Brz) delivery. The average particle size was 96.87 ± 4.43 nm, and the entrapment efficiency of the Brz was 95.41 ± 3.03%. The nanoliposomes containing TPGS (T-LPs/Brz) were compared with conventional nanoliposomes loaded Brz (LPs/Brz) and the commercial formulation AZOPT® (Brz ophthalmic suspension, Brz-Sus). Enhanced trans-corneal transport of Brz was achieved with T-LPs/Brz. Compared with Brz-Sus and LPs/Brz, the apparent permeability coefficient of T-LPs/Brz was 10.2-and 1.38-folds higher, respectively. Moreover, T-LPs/Brz extended the cornea residence of Brz. The *in vivo* studies were performed in White New Zealand rabbits treated with T-LPs/Brz had 3.1- and 1.57-folds Brz concentration 2 h after treatment than Brz-Sus and LPs/ Brz, respectively. Eye irritation experiments and histological analysis demonstrated that T-LPs/Brz had not long or short-term irritant effects and did not induce eye inflammation. Further pharmacodynamic studies showed that T-LPs/Brz maintained an adequate intraocular pressure (IOP) reduction from 3 to 11 h after administration. In comparison, Brz-Sus and LPs/Brz presented significant IOP decreases from 3–6 to 3–8 h, respectively. Moreover, they were stable for at least 10 days at 4 and 25°C. Cumulatively, the results supported the conclusion that TPGS modified nanoliposomes could be an effective delivery system for Brz to treat glaucoma.

### Stability/Shelf Life

Nanoliposome stability is an essential parameter in the physicochemical properties for subsequent exploitation as DDS. In terms of particle size. It is defined as the preservation of nanoparticle dimensionality during storage and/or an experiment. Moreover, the prospective therapeutic benefits of nanoliposomal-encapsulated drugs depend on their lifetime and distribution within the organism, which are factors related to their stability (Taira et al., 2004). The conservation of dimensionality depends on the homogeneity of the synthesized materials and stabilizing agents present during storage or use (Phan and Haes, 2019). For the above reasons, nanoliposomes should have adequate stability profile to preserve their sizes at a nanometric scale. An attractive feature of nanoliposomes is that they are metastable and can be diluted with water without changing their vesicle size distribution (Khorasani et al., 2018). Biomedical agents should be effectively cleared from the body to lower the accumulation in organs or tissues. Hence, nanoliposomes are required to have modest stability to make them more degradable and clearable, resulting in lower bioaccumulation and favorable risk–benefit ratios. The active surface of nanoliposomes may react with bioactive substances or cells in organisms responsible for initiating multifaceted reactions, ensuing the aggregation, dissolution, degradation, accumulation, and sedimentation. However, their stability not only depends on themselves but also is strongly related to their whole organized structure, the substances used to disperse or load them, the synthesis conditions, biological interactions, and other factors (Xu et al., 2018). Highlighting these considerations, studies of physical stability, and *in vitro* intestinal digestibility of nanoliposomes were evaluated (Beltrán et al., 2019). Nanoliposomes were produced by microfluidization (MF) and ultrasound (US) for high oleic palm oil (HOPO) encapsulation. The average size of nanoliposomes was 141.2 ± 1.7 to 180.0 ± 1.2 nm, having 0.141 ± 0.014 PDI for MF and 0.224 ± 0.012 PDI for the US while Zeta potential values from −45.6 ± 3.2 mV for MF and −45.9 ± 4.0 mV for the US were found. Zeta potential values were less than −30 mV being considered as coming within the range of excellent stability (Vanitha et al., 2017). No significant changes in nanoliposomes physical stability were recorded during oral phase's 2 min, the vesicle size values remained between 139.9 ± 2.1 nm and 170.0 ± 1.2 nm, with zeta potential values below −30 mV and PDI values of 0.186 ± 0.027 and 0.222 ± 0.014 for US and MF. However, both nanoliposome formulations experimented a high degree of destabilization during gastric phase. Finally, it was noted that US-prepared nanoliposomes became less digested that those prepared by MF, thereby indicating a greater stability of the US-prepared NLs, in turn enabling greater encapsulated compound protection in the gastric phase. This was also an indicator that NL encapsulation could reduce the gastric hydrolysis of HOPO and the speed at which solubilized bioactives become degraded in gastrointestinal conditions.

Bardania et al. (2017) indicated the implementation of RGD-modified nanoliposomes (RGD-MNL) for the targeted delivery of antithrombotic drug eptifibatide. The nanoliposomes were about 90 ± 10 nm in size, with an encapsulation efficiency of 37 ± 5%. The stability of nanoliposomes was evaluated by monitoring their size and drug leakage. Hence, the reported vesicle size was from 87.93 nm up to 114 nm during the storage period of 21 days at 4°C and leakage percentage values ranging from 0 to 4.5%, which indicated long term stability. According to the results, the authors concluded that the novel formulation effectively enhanced the delivery of eptifibatide to the activated platelets compared to free drugs.

Bochicchio et al. (2017) confirmed the stability of nanoliposomes loaded with a siRNA against the transcription factor E2F1 for colorectal cancer therapy. The nanoliposomes exhibited a particle size of 40 nm and high homogeneity. The spectrophotometric and electrophoretic assays corroborated the stability and 100% siRNA encapsulation efficiency. No major de-complexation of siRNA from nanoliposomes occurred following the application of an electric field; this indicated the high stability of the formed complexes. The uptake study in colon tissue cultures revealed nanoliposomes' ability to penetrate and spread all over the colon mucosa tissue. Noticeably, no evident signs of cell damage were observed, thus confirming the absence of any significant toxicity. Moreover, the nanoliposome was influential in the downregulation of the target in cultured cells and the subsequent reduction of cell growth. Finally, vital uptake and target silencing efficiencies were observed in cultured human biopsy of the colon mucosa.

## Biomedical Applications of Nanoliposomes

The term nanomedicine refers to the disease treatment, diagnosis, monitoring, and control of biological systems by using nanotechnology applications, according to the National Institutes of Health (Moghimi et al., 2005). The implementation of nanostructured systems in biomedical sciences focuses on the development of new techniques for disease diagnosis, drug design, and drug delivery particles or molecules to improve the bioavailability of a drug by subjection to suitable surface modifications where the main objective is to impart them with biological properties and functionalities (Saji et al., 2010).

**Figure 4:**
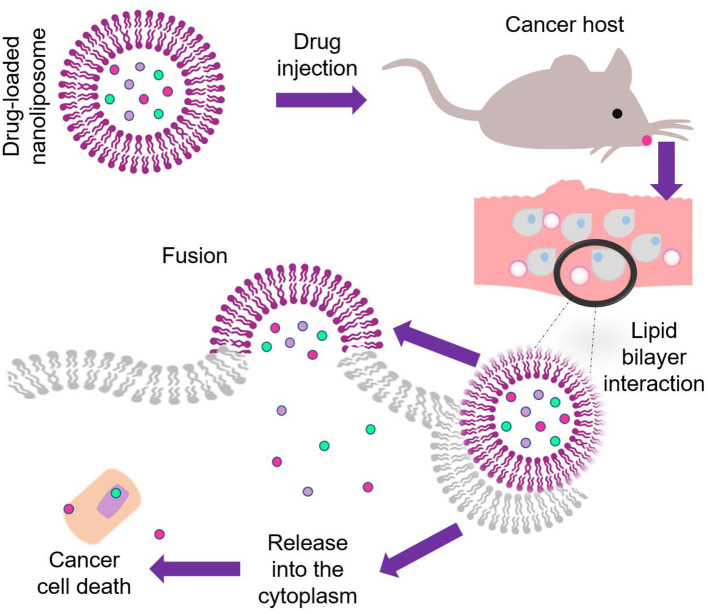
Illustration of the drug administration and release pathway of nanoliposomes against cancer cells.

Nanoliposomes have been widely studied to know their interaction effects in different strains, cultures, and animal models for the development of new drugs, vaccines, improvement of photodynamic and cancer therapy, or even as a tool for the detection of several diseases. Among the current biomedical treatments, chemotherapy sensitization of glioblastoma (75 nm) (Papachristodoulou et al., 2019), gastrointestinal disorders (145 nm) (Chen et al., 2020), cutaneous (20 nm), and fungal infections (100 nm) (Saadat et al., 2016; Bhagat et al., 2019), encapsulation of calothrixin B as anticancer agent (108 nm) (Yingyuad et al., 2018) are included as some of the successful examples of nanoliposomes as drug delivery mechanisms. Figure 4 Illustrates drug administration and release pathways of nanoliposomes against cancer cells.

### Antifungal Potentialities of Nanoliposomes

The number of cases related to superficial or systemic fungal infections has been increasing throughout the last three decades worldwide. Coupled with this, the current treatments to address these diseases are carried out during long periods and can present side effects, especially those for oral administration. Additionally, the lack of bioavailability, low penetration capacity, and poor drug release make it challenging to reach the target site (Taboada and Grooters, 2008; Kumar et al., 2014). To overcome these issues, a considerable range of nanotechnology-based products has been developed. Nanoliposomes are the most common phospholipid-based nanocarriers in dermal applications due to the high skin penetration capacity and efficacy of several drugs (Gupta et al., 2017). Amphotericin B, the first marketed product based liposomal formulation manufactured by Vestar Research Inc in 1990 (Shah and Misra, 2004). Several encapsulated substances, including commercial drugs and natural compounds, for the treatment of fungal infections, are presented in Table 4.

**Table 4:** Encapsulated substances, including commercial drugs and natural compounds, for the treatments of fungal infections.

**Drug/compound**	**Fungal infection**	**Findings**	**References**
Amphotericin B (AmB)	Systemic fungal infections and leishmaniasis	Higher accumulation values in human skin of AmB nanoliposomes and lower MIC values than as commercial product AmBisome. Deeper penetration in epithelial layers.	Perez et al., 2016
Bexarotene	Psoriasis	Reversion of psoriasis. Safety compatibility profile. Controlled release for over a period of 24 h. High percentage of entrapment efficiency.	Saka et al., 2020
Econazole	Tinea pedis	Superiority in clinical and mycological parameters of efficacy. Better tolerability compared witheconazole cream and clotrimazole cream treatment groups.	Korting et al., 1997
Fluconazole	Aspergillosis	Nano-fluconazole had better antifungal effects than the common form of drug on *A. flavus* and *A. fumigatus* species. Controlled and sustained release. Chemical stability enhancement.	Sarrafha et al., 2018
Fluconazole	*Candida albicanis*	Controlled particle size and appropriate drug loading. Superior Fluconazole entrapment and lower constant drug release compared to nanoethosome formulation. Potential application to prevent fungal biofilm formation	Zandi et al., 2018
Voriconazole (VCZ)	*Candida albicanis*	Effective, biocompatible, biodegradable and safe antifungal for intravenous delivery. Protection from premature metabolism.	Veloso et al., 2018

A mucoadhesive nanoliposomal formulation for vaginal delivery of ciclopirox (CPO) was prepared (Karimunnisa and Atmaram, 2013). The average size of nanoliposomes was found in the range of 196 ± 1.73 nm, entrapment efficiency of 44.89 ± 3.2%, and a zeta potential of −56.2 ± 1.4 mV. The antifungal activity of the CPO liposomes was confirmed against *Candida albicans* ATCC 10231 in comparison with pure CPO at pH 4.5. It was found that the pure drug showed the complete killing of Candida within 3 h as colonies were absent. At 3 h, the nanoliposomes brought about a significant reduction in the number of colonies (up to 28 ± 8) compared to its initial count (152 ± 12), whereas complete eradication was observed at the end of 6 h. The *in-vitro* antifungal activity testing concluded that CPO entrapped in nanoliposomes too demonstrated antifungal activity.

Risaliti et al. (2020) incorporated *Artemisia annua* essential oil (AEO) against *Candida* species (*C. krusei, C. parapsilosis, C. dubliniensis, C. norvegensis, C. tropicalis*, and *C. albicans*) in a nanoliposomal formulation (AEOL). Encapsulation efficiency was about 75%, and the recovery percentage was more than 90%. The nanoliposomes' performance against different *Candida* strains was assayed using the broth microdilution assay, evaluating the Minimum fungicidal concentration (MFC) (mg/ml ± SD) of AEO and AEOL prepared with RPMI-MOPS. The MFC values ranged from ca. 10 to ca. 42 mg/ml of AEO, while AEOL were tested between 5 and 10 mg/ml. Among the Candida species tested, the most susceptible to AEO was *C. norvegensis* (6.25 mg/ml), followed by *C. albicans* and *C. krusei*. In comparison, the most susceptible species to AEOL was *C. norvegensis* (5.00 mg/ml), followed by *C. krusei*. These findings suggested that AEOL could optimize biological properties and defeat fungal infections. The average MFC for AEO loaded nanoliposomes was generally one-third of AEO, demonstrating the antifungal activity enhanced by nanoliposomes. To avoid the verbose effect and unnecessary literature discussion, drug-loaded nanoliposome with antifungal attributes against various fungal strains are summarized in Table 5.

**Table 5:** Drug-loaded nanoliposome with antifungal attributes against various fungal strains.

**Fungal strain**	**Co-agent/Drug**	**Method**	**Antifungal activity**	**References**
*Candida albicans* (ATCC 10231)	CPO	Evaporation	up to 28 ± 8^a^	Karimunnisa and Atmaram, 2013
*Candida albicans*	Uconazole	Thin layer hydration method	4.0 (μ/ml)^b^	Mehrdad et al., 2016
*Candida parapsilosis*	Uconazole	Thin layer hydration method	3.0 (μ/ml)^b^	Mehrdad et al., 2016
*Candida glabrata*	Uconazole	Thin layer hydration method	8.0 (μ/ml)^b^	Mehrdad et al., 2016
*Candida krusei*	Uconazole	Thin layer hydration method	64.0 (μ/ml)^b^	Mehrdad et al., 2016
*Candida albicans*	PC:Ch:Span 60 at a molar ratio of 1:1:1	Thin film hydration	31.08 ± 1.52 (mm)^c^	Salem et al., 2016
*Candida albicans*	PC:Ch:Span 60:SA at a molar ratio of 1:1:1:0.15	Thin film hydration	34.66 ± 2.30 (mm)^c^	Salem et al., 2016
*Candida albicans*	PC:Ch:Span 60:DCP at a molar ratio of 1:1:1:0.15	Thin film hydration	29.52 ± 1.85 (mm)^c^	Salem et al., 2016
*Aspergillus niger*	PEGylated curcumin	Hydrating thin lipid film followed by sonication and extrusion	13.0 (nm)^c^	Mittal et al., 2019
*Candida albicans*	PEGylated curcumin	Hydrating thin lipid film followed by sonication and extrusion	11.5 ± 0.5 (nm)^c^	Mittal et al., 2019
*Fusarium oxysporum*	PEGylated curcumin	Hydrating thin lipid film followed by sonication and extrusion	10.5 ± 0.5 (nm)^c^	Mittal et al., 2019
*Candida parapsilosis* (ATCC 22019)	AEO	Film hydration method	>10.00 (mg/ml)^d^	Risaliti et al., 2020
*Candida krusei* (ATCC 6258)	AEO	Film hydration method	8.33 ± 2.90 (mg/ml)^d^	Risaliti et al., 2020
Candida albicans (ATCC 90028)	AEO	Film hydration method	10.00 ± 0.00 (mg/ml)^d^	Risaliti et al., 2020
*Candida glabrata* (ATCC 90030)	AEO	Film hydration method	8.33 ± 2.90 (mg/ml)^d^	Risaliti et al., 2020
*Candida albicans* (ATCC 10231)	AEO	Film hydration method	10.00 ± 0.00 (mg/ml)^d^	Risaliti et al., 2020
*Candida dubliniensis* (CBS 8501)	AEO	Film hydration method	10.00 ± 0.00 (mg/ml)^d^	Risaliti et al., 2020
*Candida krusei*	AEO	Film hydration method	7.50 ± 3.51 (mg/ml)^d^	Risaliti et al., 2020
*Candida glabrata*	AEO	Film hydration method	>10.00 (mg/ml)^d^	Risaliti et al., 2020
*Candida norvegensis*	AEO	Film hydration method	5.00 ± 0.00 (mg/ml)^d^	Risaliti et al., 2020
*Candida tropicalis*	AEO	Film hydration method	10.00 ± 0.00 (mg/ml)^d^	Risaliti et al., 2020

a *Reduction in number of colonies*,

b *MIC*,

c *Zone of inhibition*,

d *MFC*.

*MIC, Minimum inhibitory concentration; MFC, Minimum Fungicidal Concentration, AEO, Artemisia annua essential oil; SD, standard deviation; CPO, Ciclopirox olamine; PC, Phosphatidylcholine; Ch, Cholesterol; DCP, dicetyl phosphate*.

### Skin-Curative Potential of Nanoliposomes

**Figure 5:**
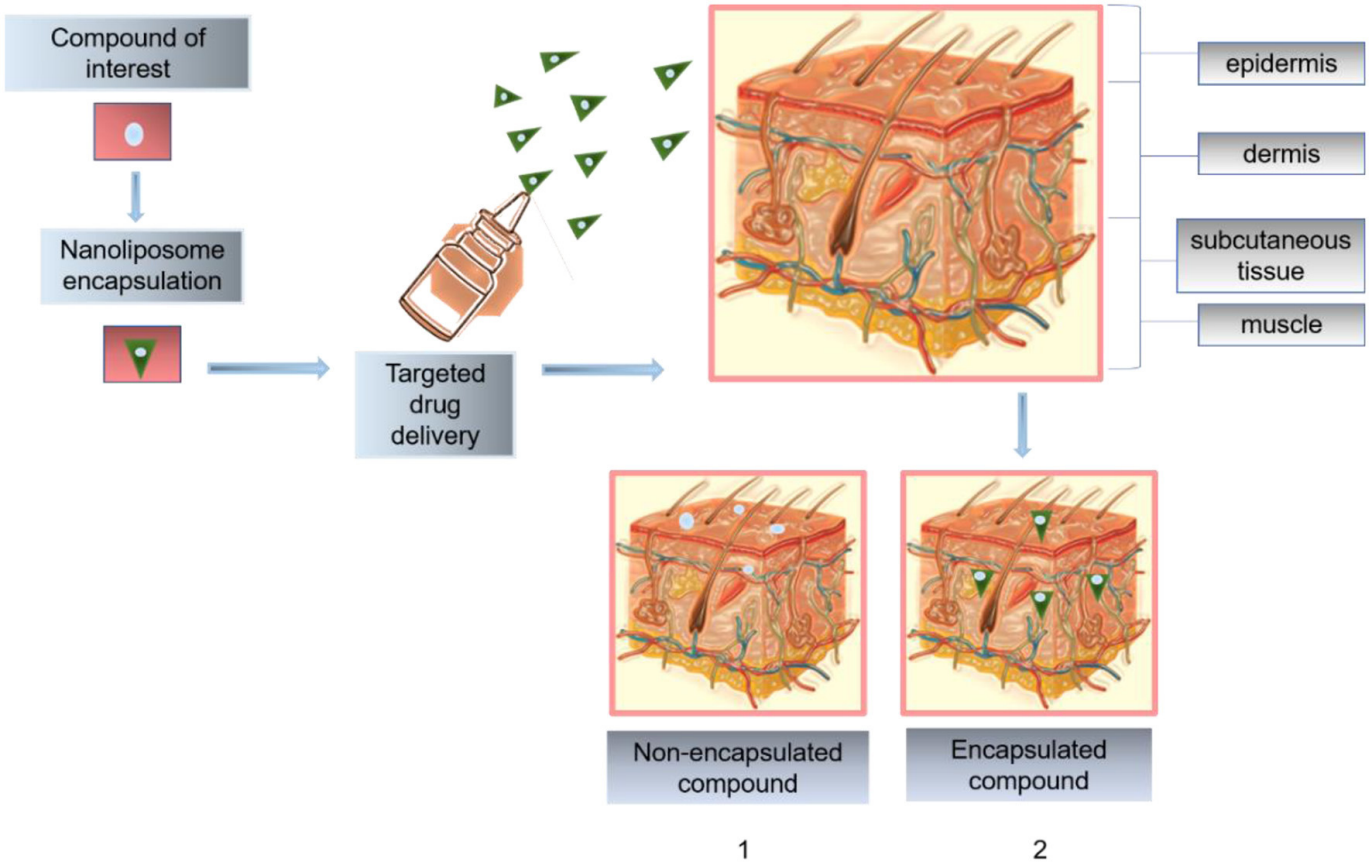
Penetration effect of a nano encapsulated compound through skin layers compared to the non-encapsulated compound. (1) shows the poor penetration effect and the lack of biodistribution across the epidermis and dermis; (2) illustrates the route of nanoliposomes into deeper skin layers.

**Figure 6:**
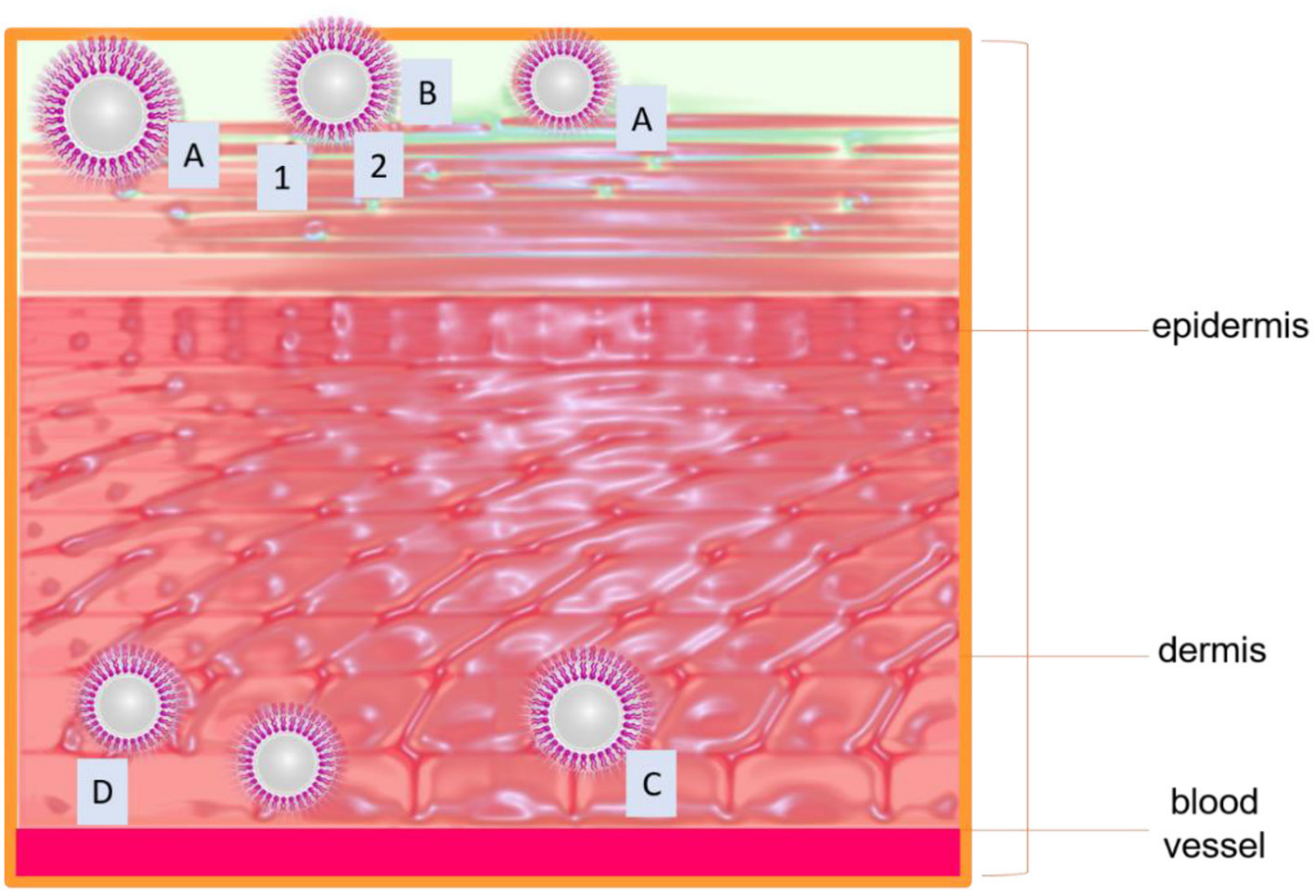
**(A)** Adsorption of liposomes to the skin surface; drug transfer from liposomes to skin. **(B)** Rupture of vesicles, the release of content, and the penetration of the free molecules into the skin via intracellular (1) or intercellular route (2). **(C)** Penetration of unilamellar vesicles via the lipid-rich channels to the dermis where they slowly release their content due to disruption or degradation of liposomal membranes. **(D)** Penetration of multilamellar vesicles via the lipid-rich channels. On the route of penetration of multilamellar vesicle can lose one or more outer lipid lamellae which would lead to partial release of the encapsulated material.

Skin is the largest and the most important organ for tropical and systemic drug administration. Its action mechanism is to protect the organism from the environment, acting as a passive barrier to the penetrant molecules. However, its exposure to the environment promotes susceptibility to damage and injury. The reason behind that common lesions is related to skin (Wang et al., 2019). Stratum corneum (SC) is the main barrier of the skin, composed of 15–20 layers of dead epidermal cells. This barrier is rich in ceramides, cholesterol, and fatty acids. With these considerations in mind, nanoliposomes become suitable for potential applications in topical drug delivery. Nanoliposomes are usually implemented as penetration enhancers of active ingredients into the skin layers. Their composition allows them to create a drug reservoir when mixing with SC lipids like ceramides, thus promoting lipophilic drug permeation of the skin (González-Rodríguez and Rabasco, 2011; Rahimpour and Hamishehkar, 2012). Figure 5 illustrates the effect of a nano encapsulated compound through skin layers compared to the non-encapsulated compound. Hasanpouri et al. (2018) evaluated nanoliposomes and nanotransferosomes in the dermal delivery of tetracycline hydrochloride (TC) for acne treatment. The particle size and distribution of TC-loaded liposomal formulation were found to be 74.8 ± 9.5 nm with a polydispersity index (PDI) 0.26 ± 0.03, while the mean zeta potential value was were 17.2 ± 5.2 mV indicating lack of colloidal stability due to was less than ± 30. However, the authors suggested the possibility of a topical aqueous gel for the final formulation dosage of the vesicular nanostructures which is in agreement with a previous study of skin-aging protection reported by Heydari et al. (2017). The *in vitro* drug release profile indicated that the percentage of released TC from liposomal formulation (55 ± 5.5%) was higher than that of transferosomal formulation (21.6 ± 4.6%), indicating maintaining the drug entrapped until its delivery to the target tissue and microorganism and preventing drug leakage, in this way its advantage in superior dermal delivery probably results in better clinical outcomes. Foldvari et al. (1990) studied the fate of liposomes loaded with lidocaine and the encapsulated drug after topical application on the skin. The investigation compared the effect of lidocaine encapsulated into liposomes or incorporated into Dermabase ® cream supplied to human volunteers about 20–25 years of age. The anesthetic effect produced by the liposome-encapsulated lidocaine was longer than the cream form and 4 h after the removal of the preparations the effect of liposomal lidocaine was still about two times greater than the conventional dosage form and the provided efficient analgesia of the intact skin was reflected in the measurement of high painless scores. It was also found a size restriction to penetration, because liposomes larger than about 0.7 μm were not observed during the electron microscopic studies. As consequence of this study, a hypothetical mechanism of interaction of topical liposomal systems with the skin was proposed for the authors and summarized in Figure 6, as follows:

- Liposomes can be absorbed to the skin surface intact before their penetration into the skin, either intercellular or intracellular journey.
- Some liposomes can rupture on the surface of the skin.
- The penetration of smaller vesicles is more probable. However, the intradermally localized uni- or oligolamellar vesicles may be derived from multilamellar liposomes, which lost their outer bilayers during penetration.

Regarding the previous model, size is crucial in the design and formulation of new drug delivery routes based on nanoliposomes for dermal and topical approaches. Thus, given the size range of nanoliposomes, they are promising candidates for implementing more realistic and functional target DDS in the treatment of skin diseases.

### Nanoliposomes Based Targeted Drug Delivery

Targeted drug delivery can be defined as a strategy that selectively and preferentially delivers the therapeutic agents or active ingredients to a target area concurrently failing access to the non-target site, thus maximizing the effectiveness of the drug (Rahimpour and Hamishehkar, 2012; Tekade et al., 2017). Nanoliposomes are considered one of the most biocompatible nanocarriers used for targeted drug delivery because of their capacity to increase the bioavailability and biodistribution of the selected encapsulated agent site by overcoming the obstacles of cellular uptake (Joshi and Joshi, 2019).

Active or triggered mechanisms can be achieved by nanoliposomes based therapy. For the first type, the nanoliposome's surface is done by ligand and antibodies, while in triggered drug delivery, the drug release is produced via stimuli sensitive (Singh et al., 2017). Internal drug triggers include pH, small biomolecules, enzyme or hormone level, glucose, or redox gradient related to the illness pathological aspects. External stimuli, including hyperthermia, ultrasound (US), light, and magnetic field, are also used to trigger the ill site's drug release. According to Darvin et al. (2019), a smart DDS can reach a particular site where the drug is intended to release. It can also release the drug in response to specific stimulations (e.g., temperature, light, ultrasound, pH, magnetic, electric field, enzyme, redox). This ability makes them intelligent systems capable of self-regulation, integrated sensing, monitoring, and activation by the environment and stimuli (Wang and Kohane, 2017).

Chen et al. (2018) synthesized a Co-delivery of doxorubicin (DOX) and imatinib (IM) by pH-sensitive cleavable PEGylated nanoliposomes with folate-mediated targeting to overcome multidrug resistance. The pH-sensitive nanoliposomes were modified with cleavable TPGS analog (mPEG2000-Hz-VES) and folate (FA-PEG3350-CHEMS) co-delivery of DOX and IM. Alpha tocopheryl acid succinate (VES) was chemically conjugated to polyethylene glycol (PEG) via acid-labile hydrazone linker at pH 7.4. The reported design allowed the folate-bond nanoliposomes to be guided to the tumor cells through the selective overexpression of folate receptors. Upon the targeted cells approaching, the pH-sensitive hydrazone bonds were hydrolyzed by the acidic environment. The nanoliposomes were fused on the tumor membrane to lead to the full drug release at pH 5–6 so that the acid-sensitive drug release profile of the nanoliposomes was controlled. Folate was used to improve tumor cell selectivity and uptake efficiency. The formulation could maintain stability in blood circulation with diameters of 100 nm, entrapment efficiency of 96.2 ± 1.4, and 96.9 ± 1.2% for DOX and IM. Simultaneously, the zeta potential and polydispersity index values were −20 ± 2 mV and 0.103 ± 0.006. Moreover, *in vitro*, pH-sensitive drug release was performed at pH 5.5, 6.5, and 7.4. The study revealed that under the acidic condition at pH 5.5 for 72 h, the releasing rate of DOX and IM from nanoliposomes increased significantly, and the cumulative release percentages of DOX and IM reached 90.73 and 92.37%. Additionally, a membrane fusion assay was performed to determined pH-triggered release, using R18 as a probe inserted into the lipid membrane followed by fluorescence self-quenching. It was observed a gradual increase in R18 fluorescence with the decreasing pH, attributed to the long PEG chain of DSPE-mPEG2000 hindered the pH-sensitive release from nanoliposomes, which composed of DOPE and CHEMS, by blocking the membrane fusion between liposomes at low pH. The designed nanoliposomes significantly enhanced anti-tumor effects both *in vitro* and *in vivo*.

A comparative study of smart ultrasound-triggered doxorubicin-loaded nanoliposomes was performed by Shalaby et al. (2020) in HeLa cells. The study compared the minimization dose of DOX and ultrasound (US) intensity in two nanoliposome systems, one of them was tailored to be responsive for US non-thermal effects (DOX-USLs), and the other was designed to be thermoresponsive (DOX-TSLs). Both systems were loaded with DOX and evaluated for *in vitro* tumor treatment and compared in terms of cellular uptake, cell viability, and apoptosis. Ultrasound- triggered the release of DOX from TSLs was conducted using a 0.8 MHz ultrasound system at an intensity of 3 W/cm^2^ while USLs at the frequency of 0.8 MHz with a power intensity of 1 W/cm^2^. The combined treatment showed markedly improved cellular uptake, tumor cytotoxicity, and enhanced apoptosis compared to free DOX treatment. A significant higher nuclear uptake and cytotoxic effect were observed from DOX-TSLs (0.1 μg/ml) compared to DOX-USLs (0.2 μg/ml), and the use of both systems had enhanced tumor apoptotic effect. The authors attributed the superior cytotoxic effect to the treatment with the US in both systems. US-mediated cavitation promoted membrane permeability and increased the intracellular accumulation of drugs. Additionally, the US also enabled the disruption of nanoliposomes, which facilitated DOX release and improved the therapeutic response.

Functional nanoliposomes have been implemented for enhanced mitochondria-targeted gene delivery and expression by Green et al. (2017), the nanoliposome formulation composed of dequalinium-DOTAP-DOPE (DQA80s) was used as a vector for target drug delivery and compared with a control vector (DQAsomes) in HeLa cells and dermal fibroblast. The developed nanoliposomes exhibited better stability than conventional transfection or mitochondrial agents, excellent potential for efficient intracellular uptake, and effective mitochondrial targeting in HeLa. The *in vitro* transfection essay suggested that DQA80s resulted in an improved transfection, with high membrane permeability able to promote the escape of the complex from the endosome compared to DQAsomes. Additionally, the complexes conformed by (DQA80s/pDNA) demonstrated higher cellular uptake, more rapid escape from endosomal membranes, and robust intra-mitochondria localization. The schematic of the formation of DQA80plexes (DQA80s/pDNA complexes) and transport of DQA80plexes to the mitochondria via the endocytosis pathway. Cho et al. (2015) confirmed the utilization of targeted fluorescent nanoliposomes to detect early cartilage damage in the post-traumatic osteoarthritis mouse model. The nanoliposomes were loaded with a fluorescent dye and conjugated to a collagen type II antibody to perform the *in vivo* study. The targeted nanoliposomes showed an affinity for homing to damaged articular cartilage due to the specific binding to degraded cartilage in a manner proportional to the degree of injury and showed a specificity of binding. Furthermore, the antibody-conjugated nanoliposomes exhibited potential as a targeted drug delivery tool to chondrocytes, also able to provide a non-invasive specific diagnostic method for detection and measurement of arthritic damage and can be intravenously administered. To avoid the verbose effect and unnecessary literature discussion, targeted drug delivery attributes of various drug-loaded nanoliposome are summarized in Table 6.

**Table 6:** Targeted drug delivery attributes of various drug-loaded nanoliposome.

**Loaded drug**	**Main target**	**Animal model**	**Drug release (%)**	**Release time (h)**	**References**
Tadalafil	Wound healing and scar formation including reepithelization and angiogenesis	Yes (Sprague–dawley female rats)	47.8 ± 5.5	24	Alwattar et al., 2020
Triptolide	Vascular endothelial cells	Yes (C57BL/6J wild-type male mice)	More than 90	480	Lai et al., 2020
Teriflunomide	Rheumatoid arthritis	Yes (Female Wistar rats)	73.21 ± 2.1	24	Mahtab et al., 2020
Bevacizumab	Ocular disorders	No	20.6 ± 2.42	40	Malakouti-Nejad et al., 2020
Sorafenib	Liver cancer therapy	Yes (Balb/c-nu mice) Nude mice bearing tumors	–	–	Ye et al., 2020
Carboplatin	Brain cancer cell lines	No	24.8	36	Hassanzadeganroudsari et al., 2019
Teriflunomide	Rheumatoid arthritis	Yes (Female Wistar rats)	85.33 ± 8.86	24	Mahtab et al., 2019
Artemether	Parenteral delivery	Yes (Swiss albino mice)	65	30	Shakeel et al., 2019
Lumefantrine	Parenteral delivery	Yes (Swiss albino mice)	51	30	Shakeel et al., 2019
Bleomycin	Tumor cells	No	34.57 ± 3.94	48	Chiani et al., 2018
Doxorubicin hydrochloride	Head and neck squamous cell carcinoma	No	84	84	Mohan et al., 2016
Resveratrol	Head and neck squamous cell carcinoma	No	Less than 80	84	Mohan et al., 2016
Topotecan	Tumor cells	Yes (NUDE-Hsd:Athymic mice)	Up to 75	96	Zucker et al., 2012
Vincristine	Tumor cells	Yes (NUDE-Hsd:Athymic mice)	Up to 60	96	Zucker et al., 2012
Doxorubicin	Specific cells or tissue targeting	Yes (rats)	69.91% ± 1.05	09	Rudra et al., 2010
Doxorubicin and Phosphatidylethanolamine	Specific cells or tissue targeting	Yes (rats)	77.07% ± 1.02	09	Rudra et al., 2010

## Toxicological Aspects of Nanostructured Systems

Throughout the last decades numerous types of nanostructured systems have been developed based on various components, including metal oxides, silica, carbon, nanocrystals, polymers, lipids, dendrimers, and quantum dots. Nanotoxicology investigates the interactions of nanostructures with biological systems (Ciucǎ et al., 2017). The biggest challenge faced by the scientific community involved in drug development is to deliver a safe and effective dosage of drugs without causing systemic toxicity (Sharma et al., 2012). For the specific case of nanoliposome formulations, they are considered as optimal carriers since phospholipids used in their preparation, such as phosphatidylcholine and phosphatidylethanolamine, are also present in natural cell membranes. However, it is imperative to consider the lipid composition and the desired application to minimize side effects. Mozafari et al. (2007) examined the cytotoxicity of anionic nanoliposomes and nucleic acids (nano lipoplexes) prepared by heating method and compared with the conventional preparation method. Cytotoxicity evaluations performed by two different assays (neutral red uptake (NRU) and 3-(4,5-dimethylthiazol-2-yl)-2,5-diphenyltetra- zolium bromide (MTT)) indicated that nanoliposomes were completely non-toxic in the cell-line tested, whereas conventional liposomes revealed significant levels of toxicity. This may be due to the presence of trace amounts of solvent applied during their preparation, which suggested further consideration of synthesis methodologies for the fabrication of nanoliposomes, mostly when organic solvents are used. These findings also indicated that nanoliposomes have great potential as non-toxic delivery vehicles in human gene therapy and drug delivery applications.

Regarding the impact of particle size on nanoliposomes' toxicity for clinical applications, particle size and size distribution are dominant factors for the stability assessment of a colloidal formulation upon storage, encapsulation efficiency, drug release profile, bio-distribution, mucoadhesion, cellular uptake, and clearance. Nevertheless, the size stability issue is more imperative for nanosystems compared to microsystems. This reason is due to the fact that DDS at the nanoscale has a larger specific surface area compared to microsystems (Danaei et al., 2018a). This would entail that more of the drug is closer to the surface of the particle compared to a larger molecule. Being at or near the surface would lead to faster drug release. Moreover, the high surface area in nanosystems such as nanoliposomes also implies that particles tend to agglomerate to minimize the energy. According to Bruinink et al. (2015), nanomaterials' agglomeration is still a controversial topic with respect to toxicity. It may be disclosed that uptake through the lung is limited to particles and agglomerates that can reach the alveolar region in the nanometer to the sub-micrometer range. Nevertheless, the incorporation of surfactants and stabilizers in nanoliposome preparation has been proposed as a good alternative to favors the electrostatic repulsion that prevents the loss of encapsulated drugs and the increase in the size of the vesicles (González-Rodríguez and Rabasco, 2011). On the other hand, one can assume that it would be advantageous to design nanoparticle systems with a large surface area to volume ratio; however, toxicity must always be tracked. The size of the nanoparticle determined the biological fate and, coupled with the PDI, are the main physicochemical attributes that influence the endocytosis-dependent cellular uptake. Cellular uptake of small molecules and particles depends mainly on endocytosis, and the two main mechanisms are reported to be pinocytosis and phagocytosis. Physiological processes such as hepatic uptake and accumulation, tissue diffusion, tissue extravasation, and kidney excretion significantly depend on particle size. In terms of nanosystems such as nanoliposomes, endothelial filtration can remove particles up to 150 nm in the liver. In contrast, particles below 10 nm can leave the systemic circulation via the lymph nodes (Psimadas et al., 2012). It has been reported that nanoparticles with dimensions of less than 5–10 nm are promptly cleared after systemic administration, whereas particles from 10 to 70 nm in diameter mostly penetrate capillary walls across the body; larger particles with dimensions of 70–200 nm regularly remain in circulation for a long period. Other reports in the literature indicate that nanosystems of less than 50 nm administered through intravenous injection reach the tissues faster than those of 100–200 nm in size and exert stronger toxic effects. If the size of the nanosystem is reduced, its contact surface will increase, and the level of oxidation and DNA damage will also rise. The size of nanoliposomes indicates their pharmaceutical behavior, that is, sizes of less than 50 nm quickly connect to all tissues and exert toxic effects. Nanoliposomes larger than 50 nm are used by the respiratory system, which stops its path to other tissues. But, organs like the liver and spleen are the main targets of oxidative stress (Ajdary et al., 2018). Moreover, the mechanism of action of the drug may vary because of the size of drug carriers. Drugs carried by micron-sized particles promote cell death mainly by necrosis, whereas nanoparticles cause cell death by apoptosis. Nanosize particles get in the cells and release the drug gradually to work on the cellular apoptotic system. However, micron-size drug carriers, because of their insufficiency of passage into the cells, could have released drugs outside the cellular environment, causing high local drug concentration, leading to cellular necrosis (Mukherjee et al., 2016). Shakeel et al. (2019) described the *in vivo* and *in vitro* evaluation of artemether and lumefantrine co-loaded nanoliposomes with the particle size of 112 nm for parenteral delivery. The toxicological examination suggested no significant evidence of renal and hepatic toxicity in tested animals. It was deduced that nanoliposomes could improve the availability of artemether and lumefantrine by prolonging drug retention *in vivo*. Yang et al. (2019) evaluated lapatinib and doxorubicin co-loaded in PEGylated nanoliposomes with an average size of 100 nm in two human lung adenocarcinoma cell lines. The formulation exhibited negligible toxicity to somatic cells, indicating the significantly reduced side effects. Besides, a decrease in toxicity was observed compared to a DOX loaded liposomal formulation and free DOX at higher concentrations. The DOX dose in the nanoliposome formulation was half of that in the comparative samples. Also, it could still maintain therapeutic efficacy and side effects reduction. On the other hand, the previous investigation accomplished by Tuerdi et al. (2016) reported the improvement of therapeutic effects of simvastatin (SMV) loaded nanoliposomes (SMV-Lipo). However, in another report published by Tuerdi et al. (2020), it was found that SMV-Lipo (121 ± 5.5 nm) induced myocardial and hepatic toxicities due to its absorption enhancement in mice. The organ toxicity was evaluated in presence and absence of isoproterenol and compared to those of free SMV. Results demonstrated that compared to free SMV, the SMV-Lipo administrated at an equal dose of 25 mg/kg/d led to severe myocardiotoxicity, hepatotoxicity at baseline and more pronounced liver injury with elevation of alanine aminotransferase. Muscular adverse effect was also observed in SMV-Lipo treated group but not in SMV group. Despite of the studies revealed that compared to free SMV, the SMV-Lipo administration significantly improved the plasma SMV concentration, and the oral bioavailability was 6.5 times of free SMV. Remarkably, when the dosage of free SMV increased to 50 mg/kg/d, yielding the comparable plasma concentration as SMV-Lipo given at 25 mg/kg/d, the myocardiotoxicity was observed in free SMV treated mice as well, which further confirmed that the enhanced absorption of SMV by the nanoliposomal formulation resulted in more severe myocardiotoxicity than the equal dose of free SMV. These findings suggest that besides particle size, toxicity must be address by considering some others physicochemical factors such as absorption capacity that affect the composition and performance of nanoliposomes for drug delivery purposes.

Besides, the implementation of analytical techniques for toxicity evaluation has allowed monitoring the *in vivo* fate of nanoliposomes. Being quantitative methods, such as fluorescence labeling, radiolabeling, magnetic resonance imaging (MRI), mass spectrometry, and computed tomography (CT) some of the most used given their specificity and excellent sensitivity (Rizvi and Saleh, 2018; Su et al., 2018). However, further *in vitro* and *in vivo* research under different conditions is still necessary to evaluate the toxicity of nanoliposomes prior to clinical applications.

## Compatibility—Patient Compliance and Safety

Drugs-based liposomes have already been successfully tested in humans and approved by FDA, examples include DepoDur ®, Lipusu ®, Exparel, among others (Beltrán-Gracia et al., 2019). Exparel ® is a bupivacaine liposome injectable suspension (3,000−30,000 nm) developed by Pacira Pharmaceuticals®, Inc. and approved in 2011 by FDA. The suspension is indicated for postoperative pain after hemorrhoidectomy and bunionectomy. Mont et al. (2018) compared the effects of local infiltration analgesia (LIA) with liposomal bupivacaine (LB) in patients undergoing total knee arthroplasty (TKA) where a total number of 140 patients, including adult men and non-pregnant women were randomized to LIA with LB 266 mg/20 ml (admixed with bupivacaine HCl 0.5%, 20 ml) or LIA with bupivacaine HCl 0.5%, 20 ml. Standardized infiltration techniques and a standardized multimodal pain management protocol were used. The coprimary efficacy endpoints were area under the curve (AUC) of visual analog scale pain intensity scores 12–48 h (AUC12-48) post-surgery and total opioid consumption 0–48 h post-surgery. Results showed that an opioid-sparing multimodal pain management approach using LIA with LB could safely manage pain while further reducing or eliminating the need for opioids following TKA, which also could have a. In this setting, LIA with LB significantly improved postsurgical pain, opioid consumption, and time to first opioid rescue, with more opioid-free patients and no unexpected safety concerns.

Concerning the clinical trials with formulations based-nanoliposomes, one of the most recent drugs-based approved by the FDA in 2017 is VYXEOS (100 nm), a combination of daunorubicin-cytarabine developed by Jazz Pharmaceuticals, Inc. (Beltrán-Gracia et al., 2019) for the treatment of adults with newly diagnosed therapy-related AML (t-AML) or AML with myelodysplasia related changes (AML-MRC), two types of AML having a poor prognosis, being the first FDA-approved treatment for this specific type of sickness (FDA, 2017). For the clinical phase 3 test, 309 patients 60–75 years of age with newly-diagnosed t-AML or AML-MRC through a randomized (1:1), multicenter, open-label, and active-controlled trial study comparing VYXEOS to a classic combination of daunorubicin and cytarabine (7+3) administrated intravenously, where it was demonstrated that VYXEOS had an estimated median overall survival of 9.6 months compared with 5.9 months for the 7+3 control (hazard ratio 0.69; 95% CI: 0.52, 0.90; *p* = 0.005). Moreover, VYXEOS nanoliposomes exhibited a prolonged plasma half-life following intravenous infusion, with greater than 99% of the daunorubicin and cytarabine in the plasma remaining encapsulated within the nanoliposomes, which accumulate and persist in high concentration in the bone marrow, where they are preferentially taken up intact by leukemia cells in an active engulfment process (Jazz Pharmaceuticals UK, 2019).

Another drug tested and approved by FDA is AmBisome® (Gilead Sciences, 2012). AmBisome ® is a nanoliposome formulation with a reported vesicle size of 45–80 nm, administrated by intravenous fusion. The formulation is indicated for empirical therapy for presumed fungal infection in febrile, neutropenic patients. Eleven clinical studies were conducted to support its efficacy and safety in patients with Aspergillus species, *Candida* species and/or *Cryptococcus* species infections and visceral Leishmaniasis. These patients either had fungal infections refractory to amphotericin B deoxycholate, were intolerant to the use of amphotericin B deoxycholate, or had pre-existing renal insufficiency. Patient recruitment involved 140 infectious episodes in 133 patients, with 53 episodes evaluated for mycological response and 91 episodes assessed for clinical outcome. Clinical success and mycological eradication occurred in some patients with documented aspergillosis, candidiasis, and cryptococcosis. Regarding the treatment of Leishmaniasis, AmbiSome ® achieved high rates of acute parasite clearance when total doses of 12–30 mg/kg were administered in immunocompetent patients. Most of these immunocompetent patients remained relapse-free during follow-up periods of 6 months or longer. While acute parasite clearance was achieved in most of the immunocompromised patients who received total doses of 30–40 mg/kg, most of these patients were observed to relapse in the 6 months following the completion of therapy. When followed for 6 months or more after treatment, the overall success rate among immunocompetent patients was 96.5%, and the overall success rate among immunocompromised patients was 11.8% due to relapse in most patients. There are no data documenting the efficacy or safety of repeat courses of AmBisome or maintenance therapy with this drug among immunocompromised patients.

Onivyde, also known as MM-398 or PEP02, is a nanoliposomal formulation of irinotecan (88~95 nm) in diameter (Drummond et al., 2006) which has demonstrated promising anticancer activity across a broad spectrum of malignancies, including pancreatic cancer, esophagogastric cancer, and colorectal cancer (Zhang, 2016). The nanoliposomal formulation of irinotecan occupies a modified gradient-loading method using sucrose octasulfate with unparalleled drug-loading efficiency and *in vivo* drug stability. A phase I study carried out by Chang et al. (2015) reported the dose limiting-limiting toxicity (DLT), maximum tolerated dose (MTD), and pharmacokinetics (PK) of PEP02 in patients with advance refractory solid tumors. It was found that myelosuppression and diarrhea were the major DLTs, and 120 mg/m^2^ was defined as the MTD. Pharmacokinetic analysis displayed that the release of free-form irinotecan from the nanoliposomes occurred slowly over time, the toxicity pattern was comparable with that of free-form irinotecan. Moreover, encouraging antitumor activities were noticed in patients who were refractory to available treatments. Furthermore, according to the database available on the FDA website, exist a variety of orphan drugs-based liposomes that have been approved for their commercial distribution and are summarized in Table 7.

**Table 7:** Orphan products-based liposomes designated and/or approved by FDA, US Food and Drug Administration. https://www.fda.gov/(accessed on 30/04/2020).

**Generic name**	**Trade name**	**Designated indication**	**Marketing approved indication**	**Sponsor**
Doxorubicin HCL liposome injection	Doxil	Multiple myeloma	Patients with multiple myeloma.	Johnson and Johnson Pharmaceutical Research and Dev.
Amphotericin B lipid complex	Abelcet	Invasive fungal infections.	Patients intolerant to conventional amphotericin B therapy.	Liposome Company, Inc.
Liposomal amphotericin B	AmBisome	Cryptococcal meningitis.	Treatment of cryptococcos	Bristol-Myers Squibb Pharmaceutical Research Institute
Liposomal amphotericin B	AmBisome	Visceral leishmaniasis.	Treatment of visceral leishmaniasis.	Fujisawa USA, Inc.
Daunorubicin citrate liposome injection	DaunoXome	HIV-associated Kaposi's sarcoma.	Advanced, HIV related Kaposi's sarcoma.	NeXstar Pharmaceuticals, Inc
Antihemophilic factor with liposome diluent	Kogenate(r) FS	Hemophilia A	N/A	Bayer HealthCare LLC
Cytarabine liposome	DepoCyt	Gliomas	N/A	Bruce Frankel, MD
Cytarabine: daunorubicin liposome injection	N/A	Myeloid leukemia	N/A	Celator Pharmaceuticals, Inc.
Gentamicin liposome injection	Maitec	Mycobacterium avium-intracellulare infection.	N/A	Liposome Company, Inc.
Liposomal nystatin	Nyotran	Invasive fungal infections.	N/A	The University of Texas
Cisplatin in liposomal formulation	SLIT Cisplatin for inhalation	Osteogenic sarcoma metastatic to the lung	N/A	Transave, Inc.
Doxorubicin liposome	Doxil	Ovarian cancer	Refractory disease to paclitaxel- and platinum	Alza Corporation
Cytarabine liposomal	DepoCyt	Neoplastic meningitis	Intrathecal treatment of lymphomatous meningitis	Pacira Pharmaceuticals, Inc.
Adeno-associated vector lipoprotein lipase protein	N/A	Lipoprotein lipase deficiency	N/A	Amsterdam Molecular Therapeutics BV
Amphotericin B lipid complex	Abelcet	Invasive protothecosis, sporotrichosis, coccidioidomycosis, zygomycosis and candidiasis	N/A	The Liposome Company, Inc.
GNE Lipoplex	N/A	Hereditary inclusion body myopathy-2	N/A	Gradalis, Inc.
HLA-B7/Beta2M DNA Lipid (DMRIE/DOPE) Complex	Allovectin-7	Metastatic melanoma (Stages II, III, and IV).	N/A	Vical Incorporated
Liposomal cisplatin	LipOva-Pt	Ovarian cancer	N/A	Transave, Inc.
Liposomal cyclosporin A	Liposomal cyclosporin A	Lung allograft and pulmonary rejection	N/A	Vernon Knight, M.D.
Liposomal N-Acetylglucosminyl-N-Acetylmuramly-L-Ala-D-isoGln-L-Ala -gylcerolidpalmitoyl	ImmTher	Osteosarcoma Ewing's sarcoma	N/A	Endorex Corp.
Liposome encapsulated recombinant interleukin-2	N/A	Brain and central nervous system (CNS) tumors. Cancers of kidney and renal pelvis	N/A	Oncothyreon Canada, Inc
Liposomal-cis-bis-neodecanoato-trans-R, R-1,2-diaminocyclohexane-Pt (II)	Aroplatin	Malignant mesothelioma.	N/A	Antigenics Incorporated
Bupivacaine liposome injectable suspension	Exparel	Nerve Block for Regional Analgesia	N/A	PaciraPharmaceuticals, Inc.

## Limitations, Research Gaps, and Challenges

Despite the significant advantages of nanoliposomes used in biomedical applications, further research is needed to improve their storage stability and overall efficacy. This typical behavior is attributed to the small vesicle size and high surface energy, which entails larger van der Waals attraction forces, thus, promoting high attraction among nanoliposomes (Dahman, 2017). Several research groups have been proposed alternatives to overcome these issues, such as the addition of surfactants or polyols to stabilize the nano liposomal suspensions (Mortazavi et al., 2007; Ebrahimifar et al., 2017; Eh Suk and Misran, 2017).

Additionally, bilayer fusion and drug leakage in nanoliposomes are other issues in terms of physical stability, which can entail a low yield of shelf life of nanoliposomes, affecting the low reproducibility and stability of nanoliposomes. However, when a nanoliposomal formulation is well-prepared, fusion is not common over time (Nounou et al., 2008; Wang et al., 2019). Besides, future research focused on cytotoxicity and *in vitro* essays must guarantee that topical and oral administration will have adequate stability and sustained drug release profiles for practical use and not only under ideal experimental conditions (Mordorski et al., 2016). Finally, the enhancement of the fabrication techniques is another challenge that research groups must attend by developing process with high scalability, reproducibility, and economically viable, also including more responsible practices with the environment that can be achieved by avoiding or reducing the use of organic solvents and detergents, as well as the incorporation of materials such as phospholipids and excipients that are in accordance with the purpose of the final product (Danaei et al., 2018b).

## Concluding Remarks and Outlook

Nanoliposomes display a broad field of nanomedicine opportunities, from their implementation as diagnostic tools until drug carriers of topical and systemic treatments. Overall they hold great potential to innovate the area of drug delivery. Therefore described, nanoliposomes have also been satisfactorily used in drug improvement of vaccines and for the treatment of several fungal and bacterial infections, inflammation, and anticancer agents regarding their multiple utilitarian properties such as large surface as a consequence of their small size. Moreover, nanoliposomes' ability to permeate skin and blood barriers entails a better sustained-release activity and selective accumulation of active compounds within tissues providing accuracy in drug targeting. Nevertheless, nanoliposomes' broad spectrum of benefits still lacks human clinical trials and more efficient fabrication techniques, which are necessary to ensure scalability and translatability from the laboratory conditions to marketed products. Additional studies are needed to explore significative risks for toxicity and implement regulations for responsible management, storage, and waste disposal during the fabrication processes.

## Author Contributions

KA-P and HI: conceptualization. KA-P and JA-C: writing—original draft preparation. DM, RP-S, and HI: writing—review and editing. HI: supervision. All authors contributed to the article and approved the submitted version.

## Conflict of Interest

The authors declare that the research was conducted in the absence of any commercial or financial relationships that could be construed as a potential conflict of interest.
